# The Western Lancet

**Published:** 1846-01

**Authors:** 


### THE WESTERN JOURNAL.

New Series.—Vol. V.—No. I.

LOUISVILLE, JANUARY 1, 1846.

### THE WESTERN LANCET.

We are grieved to see that a remark made by us, in our obituary notice of the late Professor Richardson, has provoked the displeasure of our neighbor of the Western Lancet. The editor, in his December number, after quoting from our article the words which follow: "but Dr. Richardson, while he was sensible of the superior advantages which this school (the Louisville Medical Institute) would enjoy, made up his mind to remain in the institution which he had been instrumental in building up;" goes on to express "deep regret," that they should have found a place "in *such* a journal, on *such* an occasion." They "explain," in his opinion, no part of the "history" of the deceased, have "no relationship to his character" as a public man, and must, therefore, he insists, have been "introduced for the purpose of injuring a rival medical school." He adds: "It will certainly be regarded as a unique specimen of ill-feeling, which would introduce an *electioneering* paragraph, into an obituary notice of a lamented member of the profession, and a former colleague."

Our neighbor's sensibilities are, evidently, very much shocked; he looks upon our harmless paragraph as something near akin to sacrilege. But we assure him, that we were not prompted by any

"ill-feeling" in writing it, and that nothing was farther from our expectations, than that we should offend any one by adverting to a fact, as well known as any other, in the history of Professor Richardson. As to its relevancy or importance, we are sorry that the editor of the Lancet does not think with us; but in regard to that we had to judge for ourselves; and if it occurred to us as a very momentous incident in the life of the deceased, we venture to believe that it did not appear less so to himself and his friends. Professor Richardson was the first individual, connected with the Lexington school, to broach to an influential Senator in the Kentucky Legislature the project of removing that school to Louisville; and that he continued to be one of the warmest advocates of that measure for a long time afterwards, is as certain as anything can be rendered by human testimony. It rests, in truth, upon nothing less than his own declaration to the Board of Trustees of Transylvania University, and if anything further were needed, we might add, upon the published evidence of four of his colleagues. But it is unnecessary to insist upon the truth of our statement, since the editor of the Lancet does not call it in question; he contents himself with remarking—

"In relation to the assertion contained in the obnoxious paragraph, we can only say, that we know not what views Prof. Richardson may have expressed to the writer; but this we do know, that during our short association with him, he always *decidedly* and *emphatically* expressed the contrary opinion."

It appears, then, that Prof. R. not only made up his mind to remain in Lexington, but changed his views in respect to the great superiority of Louisville over that city as a seat for a medical school; and to show, we suppose, that such changes of opinion are not rare among distinguished teachers, the Lexington editor copies largely from the discourse of our colleague and friend, Prof. Caldwell, on the "*impolicy of multiplying schools of medicine.*" He does our venerable associate the honor to say, that he "is justly regarded as one of the ablest men of our country," and that he had enjoyed "opportunities of becoming perfectly familiar with the facilities for instruction in the large cities of the United States and Europe." The "opinions" of Prof. C., to which the editor of the Lancet "concedes great weight," were, that hospitals were of no benefit to a large class; that living was "fifty per cent. higher in Louisville and

Cincinnati than in Lexington;" and that these cities afforded to pupils no superior facilities for prosecuting practical anatomy. These were the chief; to which we might reply, after the manner of our neighbor, that our learned colleague *does not entertain them now;* but since our brother editor is disposed to allow them "*great weight,*" we will state a few reasons why we are compelled to dissent from them.

Concerning hospital instruction as formerly administered, the sentiments of Prof. Caldwell were no doubt correct. A large class crowded around the beds of the sick are in no situation to see or hear to advantage; but our neighbor understands that things are ordered differently now; and that the clinical remarks and examinations made in amphi-theatres connected with hospitals, with patients full in view of all the class, surpass in interest and instructiveness any other lectures delivered on the practice of physic.

In respect to the cost of living, our colleague was altogether mistaken; experience has proved that it is not greater in Louisville than in Lexington.

The only other question relates to the comparative means of large and small cities for sustaining the anatomical department of a medical school, and we have seen that the author of the discourse referred to did not concede to the former any superiority on that point. This was his opinion, weighty or otherwise, and he expressed it with great earnestness. In his eloquent discourse, in passages quoted and apparently adopted by the Lancet, he depicts, in frightful colors, the difficulty of procuring subjects in large cities—a difficulty, which, as he forcibly expressed it, "has led to the atrocious practice of assassinating individuals, and selling their bodies to surgeons and pupils!" This appeared unanswerable, at the time; the fact seemed to be made out, that crowded cities were *not* the places to study anatomy; but it was still an opinion merely. We have had experience since, and, as a part of it, we declare to the editor of the Lancet, that "*Burking*" is still unknown in Louisville, notwithstanding that large classes have been engaged in the dissection of human subjects, every winter, for seven years past. Dr. Bush, the then Demonstrator of Anatomy, stated before the Trustees of Transylvania University, in March, 1837, that there had been, in the anatomical rooms under his supervision, *eleven* subjects during the preceding winter; and that, out of a class of two hundred and forty-two,

*one* student had engaged in dissection. The session in the Medical Institute is now just half over, and, according to information derived from the Demonstrator of Anatomy, the dissecting classes, amounting to upwards of thirty, and embracing one hundred and eighty-four pupils, have already been supplied with subjects. With the beginning of the year his first class was invited to recommence their labors, and, if his success should not be less than it has been in former years, he expects to see every class in his rooms a second time before the first of March. This is experience *versus* opinion.

It appears from the article in the Lancet, that both Dr. Richardson and Dr. Caldwell changed their ground respecting the superior fitness of Louisville for supporting a medical school. Dr. Caldwell, at one time, seems to have doubted it; but finally his doubts gave way, and he became an advocate for the removal. With Dr. Richardson the process was the reverse of this; he was the first to propose measures for transferring the medical department of Transylvania to Louisville; but, according to the Lancet, towards the close of his life he was "decidedly and emphatically" of opinion that Lexington was the better site. Here, again, we have been taught by experience. Dr. Richardson lived to see the Louisville Medical Institute numbering two hundred and eighty-six students, while he was lecturing in Lexington to a class of one hundred and fifty-six. Dr. Caldwell still lives; he does not know what the present size of the Lexington class is, but he has the satisfaction of seeing now assembled in the school of which he was the founder a class of three hundred and forty-five. These facts we hold to be more decisive of the question than any man's opinions.

And here we dismiss the subject, we trust, forever. We hope our explanation will be satisfactory to our contemporary. He cannot feel a greater repugnance to professional bickerings than we do; and again we must express our unfeigned sorrow that anything from our pen should have drawn down from him upon us so grave a lecture. We are most happy when our editorial labors meet with the approval of our readers, and although we are not so sanguine as to expect ever to please all tastes, we do hope, henceforth, to be able to "plod" along our "weary way" without giving offence to our neighbor of the Lancet. Y.

